# Rostro-caudal different energy metabolism leading to differences in degeneration in spinal cord injury

**DOI:** 10.1093/braincomms/fcab058

**Published:** 2021-03-28

**Authors:** Yuichiro Ohnishi, Masamichi Yamamoto, Yuki Sugiura, Daiki Setoyama, Haruhiko Kishima

**Affiliations:** 1 Department of Neurosurgery, Osaka University Medical School, Osaka, Japan; 2 Department of Research Promotion and Management, National Cerebral and Cardiovascular Center, Osaka, Japan; 3 Department of Biochemistry, Keio University School of Medicine, Tokyo, Japan; 4 Department of Clinical Chemistry and Laboratory Medicine, Graduate School of Medical Sciences, Kyushu University, Fukuoka, Japan

**Keywords:** ATP, degeneration, metabolism, glycolysis, oxidative stress

## Abstract

Spinal cord injury gradually spreads away from the epicentre of injury. The rate of degeneration on the rostral side of the injury differs from that on the caudal side. Rostral degeneration is an immediate process, while caudal degeneration is delayed. In this study, we demonstrated that the rostro-caudal differences in energy metabolism led to differences in the spread of degeneration in early thoracic cord injury using *in vivo* imaging. The blood flow at the rostral side of the injury showed ischaemia-reperfusion, while the caudal side presented stable perfusion. The rostral side had an ATP shortage 20 min after spinal cord injury, while the ATP levels were maintained on the caudal side. Breakdown products of purine nucleotides were accumulated at both sides of injury 18 h after spinal cord injury, but the principal metabolites in the tricarboxylic acid cycle and glycolytic pathway were elevated on the caudal side. Although the low-ATP regions expanded at the rostral side of injury until 24 h after spinal cord injury, the caudal-side ATP levels were preserved. The low-ATP regions on the rostral side showed mitochondrial reactive oxygen species production. Administration of 2-deoxy-d-glucose as a glycolysis inhibitor decreased the caudal ATP levels and expanded the low-ATP regions to the caudal side until 24 h after spinal cord injury. These results suggest that deficits in the glycolytic pathway accelerate the caudal degeneration, while immediate rostral degeneration is exacerbated by oxidative stress in early thoracic cord injury.

## Teaser

Rostral degeneration of the spinal cord injury is an immediate process, while caudal degeneration is delayed. Ohnishi et al. reported that the glycolytic pathway is involved in the delayed caudal degeneration, while immediate rostral degeneration is exacerbated by oxidative stress in early thoracic spinal cord injury.

## Graphical Abstract

**Graphical Abstract:**
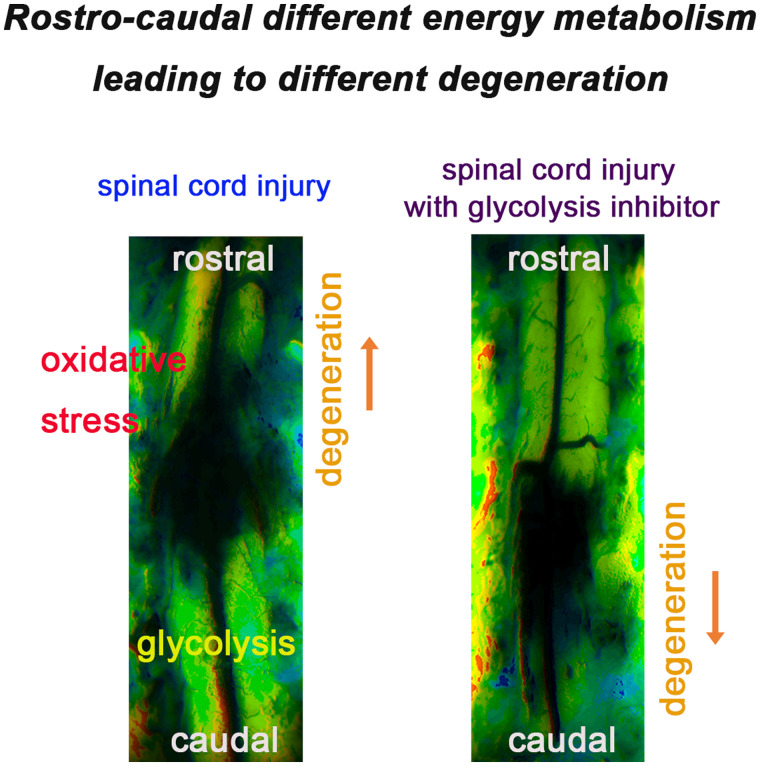

## Introduction

Spinal cord injury (SCI) is widely thought to gradually spread into adjacent uninjured tissue along with subsequent inflammation and moves in a direction away from the epicentre of injury.^1^ The rate of degeneration on the rostral side of the injury differs from that on the caudal side. In SCI, rostral degeneration is an immediate process, while caudal degeneration is delayed.^2–4^

ATP functions as a cellular energy molecule in living cells^5^ and a second messenger in extracellular spaces.^6^ Equilibrium between the consumption and production of ATP maintains cellular activity. Intracellular ATP levels have been implicated as a determinant of the cell’s decision to die by apoptosis or necrosis.^7^ Peritraumatic extracellular ATP reportedly led to irreversible increases in cytosolic calcium and cell death.^3^ However, whether or not energy metabolism is involved in the slow caudal degeneration of SCI remains unclear.

In the present study, we demonstrated that the rostro-caudal differences in energy metabolism led to differences in the spread of degeneration in early thoracic SCI using *in vivo* imaging. The blood flow and intracellular ATP were monitored in real-time. We explored the rostro-caudal energy metabolism in early SCI. These results suggest that deficits in the glycolytic pathway accelerate the caudal degeneration, while immediate rostral degeneration is exacerbated by oxidative stress in early thoracic SCI.

## Materials and methods

### Animals

Eight-week-old male ICR mice were obtained from Japan SLC, Inc. (Hamamatsu, Japan). The GO-ATeam2 Tg mice were provided by Dr M. Yamamoto. All procedures were performed in accordance with the guidelines of the Laboratory Animals Care and Use Committee (No. 29-025-012). Efforts were made to minimize the number of animals used and to limit their suffering.

### SCI model

Mice were anaesthetized with 4% isoflurane, and the anaesthesia was maintained with 2–2.5% isoflurane in air. Throughout the surgical procedures and experiments, the rectal temperature was maintained at 37.5°C using a thermostatically controlled heating pad. We palpated the 13th rib externally and then visualized this vertebra with forceps. Once landmarking at T13 had been definitively performed, we counted up to T9 from T12. Although the site of clipping was set at the T10 level, we performed opening at T9–T12 in order to visually recognize the T10 level. Therefore, using an operating microscope, laminectomy was performed at the T9–T12 thoracic vertebrae. The dorsal surface of the dura mater was exposed. SCI was induced using a disposable straight clip (AM-1: 30 g/mm^2^ of clipping power, 2-mm clip width; Natsume Seisakusho, Tokyo, Japan). Clipping and releasing were performed gently. The clip was applied vertically. The duration of compression was 15 s. The clip was limited to use in three experiments to prevent deterioration of the clip quality. All mice received subcutaneous gentamicin (8 mg/kg) and meloxicam (5 mg/kg) after the procedures. One neurosurgeon (Y.O.) who was familiar with the experimental procedures performed the microscopic surgeries. The timekeeper informed the surgeon about the timing of the clip release. We confirmed that these were reproducible SCI models regarding the locomotor function and histology.

### The locomotor function

The locomotor function of the hind limb was assessed every week (0–8 weeks) after SCI using an open-field walking test. Two examiners who were blinded to the treatments assessed the hind limb movements, and the locomotor function was evaluated according to the Basso Mouse Scale [with scores ranging from 0 (paralysis) to 9 (normal gait)].

### Histology

Mice were deeply anaesthetized, and their tissues were fixed via transcardial perfusion with 100 ml of phosphate-buffered saline (PBS; Nippon Gene Co., Ltd, Tokyo, Japan), followed by 4% PFA (FUJIFILM Wako Pure Chemical Corporation, Osaka, Japan) or 2.5% glutaraldehyde (Nacalai Tesque, Inc., Osaka, Japan). The spinal cord was embedded in OCT Compound (Tissue Tek; Sakura Finetek Japan Co., Ltd, Tokyo, Japan), and 10-μm thick sections were cut from the blocks using a cryostat (CM1510S; Leica Microsystems, Wetzlar, Germany). The sections were stained with haematoxylin and eosin and were assessed using images captured with a microscope. Tissues were fixed with 2.5% glutaraldehyde for 2 h. The samples were washed more than 3 times for 10 min each in 0.1 M phosphate buffer and then postfixed in 1% osmium tetroxide for 90 min at 4°C. Next, the samples were washed 3 times in distilled water for 10 min each. After en bloc staining in 0.5% uranyl acetate in distilled water for 4 h, the samples were washed 3 times in distilled water for 10 min each. The samples were then dehydrated using serial dilutions of ethanol (50%, 80%, 90%, 95%, and 100%) for 10 min each and treated twice with propylene oxide for 20 min each before being impregnated with 50:50 propylene oxide: epoxy resin overnight at room temperature. Finally, the samples were impregnated with 100% epoxy resin, embedded in molds, and incubated for 48 h at 60°C. Semithin sections (1 µm) were then cut using an ultramicrotome (Reichert Ultracut E, Wetzlar, Germany) and stained with 0.5% toluidine blue. The sections were assessed using light microscopy. A 100× oil immersion lens was used to calculate the g-ratios used Fiji.^8–10^

### Real-time monitoring of the blood flow

The blood flow distribution in the spinal cord was assessed using laser speckle flowgraphy (LSF), a technique used to analyse laser scattering phenomena (*n* = 4). The exposed dura was illuminated with a laser light (OZ-1; Omegawave, Inc., Tokyo, Japan) to produce a speckle pattern that was detected with a charge-coupled device (CCD) camera (ZM10-18; Omegawave, Inc.). Raw speckle images were obtained with a speckle contrast imaging software program (Laser Speckle Blood Flow Imager; Omegawave, Inc.). LSF was used to assess changes in the blood flow at a rate of 1.4 maps/s. The blood flow distribution appears in a 2D colour code map. The image and video movie files were generated via the reconstruction of speckle contrast images using an imaging software program (Laser Image Analysis Software; Omegawave, Inc.). To investigate the spatial and temporal patterns of blood flow, 11 regions of interest (ROI; 0.5 mm in diameter, round circle) were equally distributed in the rostro-caudal directions at 1-mm intervals in the spinal cord, along the posterior longitudinal vein to avoid the vessels. The ratio of blood flow was calculated by assessing the blood flow 5-mm rostral from the epicentre, and the values obtained were used as controls.

### Real-time monitoring of intracellular ATP levels

The intracellular ATP levels in GO-ATeam2 mice with normal spinal cord (*n* = 5), spinal cord compression (*n* = 6), and cardiac arrest (*n* = 2) were assessed. The Go-ATeam2 mice expressed a fluorescence resonance energy transfer-based ATP biosensor with orange fluorescent protein (OFP) and green fluorescent protein (GFP). 2-DG (Funakoshi Co., Ltd, Tokyo, Japan) was diluted in PBS and intraperitoneally administered (500 mg/kg body weight) every 6 h from 48 h before SCI to 24 h after SCI. Spinal cords were examined using a Leica M165 FC stereomicroscope (Leica) with a 2.0× objective (Plan Apo 1.0; Leica), and the following DualView2 filter sets (INDEC Biosystems, Toronto, Canada) were used: 470/40 excitation filter for dual emission ratio imaging of Go-ATeam2 and a dichroic mirror 540 DCLP of 515/30 and 575/40 for GFP and OFP, respectively. Fluorescence emission in the Go-ATeam2 mice was captured using an ORCA-Flash4.0 (Hamamatsu Photonics K.K., Shizuoka, Japan). The exposure time was 1.5 s for both GFP and OFP images. To investigate the spatiotemporal patterns of the changes in ATP levels, 11 ROIs was applied, as in LSF. The imaging data of the OFP/GFP emission ratios were analysed using MetaMorph (Molecular Devices, San Jose, CA, USA). The average fluorescence resonance energy transfer signals at the chosen ROI were calculated, and the OFP/GFP ratio obtained was applied to the following equation: fluorescence resonance energy transfer ratio=1.52×[ATP]^1.7^/([ATP]^1.7^+2.22)+0.44.^11^ Our ATP imaging approach using transgenic mice expressing ATP probes enabled monitoring of the ATP concentration in the cytosol. Control fluorescence images were obtained and used to subtract the background signal.

### Mass spectrometry

Mice with spinal cord compression were assessed (*n* = 4). SCI was induced using a disposable straight clip (AM-1; Natsume Seisakusho) for 15 s. At 20 min after SCI, mice were anaesthetized and sacrificed. Spinal cords with spinal bones were quickly removed and immediately immersed in liquid nitric oxide. Thin sections (8 μm thick) were cut with a cryomicrotome (CM3050; Leica Microsystems). To prepare thin slices of the entire spinal column, Kawamoto’s method, which preserves the morphology of the soft and hard tissues during sectioning, was used.^12^ The sections obtained on Kawamoto’s film were fixed on indium-tin-oxide-coated glass slides (Bruker Daltonics, Billerica, MA, USA) using double-sided conductive adhesive tape to facilitate electrical conduction.^12^ The sections were then spray-coated with 2,5-dihydroxybenzoic acid as a matrix (40 mg/ml, dissolved in 80% methanol) using an artistic airbrush (Procon Boy FWA Platinum 0.2-mm calibre airbrush; Mr Hobby, Tokyo, Japan). Imaging measurements were performed using an orbitrap mass spectrometer (QExactive Focus; Thermo Fisher Scientific, Waltham, MA, USA) coupled with an atmospheric pressure-scanning microprobe matrix-assisted laser desorption/ionization ion source (AP-SMALDI10; TransMIT GmbH, Giessen, Germany). The raster step size was set at 50 μm. Signals within a mass range of 80–900 were acquired with a mass resolving power of 70 000 at m/z 200. Thereafter, the spectral data were transformed to image data and analysed using the ImageQuest 1.0.1 (Thermo Fisher Scientific) and SCiLS 2019a (Bruker Daltonics) software programs.

### Measurement of the principal metabolites in the glycolytic pathway, TCA cycle, and amino acids

Mice with spinal cord compression (*n* = 5) and laminectomy only (*n* = 4) were assessed. SCI was induced using a disposable straight clip (AM-1; Natsume Seisakusho) for 15 s. At 18 h after SCI and laminectomy, we euthanized the mice, dissected the spinal cords with bones, and flash-froze the spinal cord in liquid nitrogen at −80°C. The spinal cord was dissected into rostral and caudal segments, each of which was 4 mm in length (between 2 and 6 mm from epicentre). Metabolites in the glycolytic pathway, TCA cycle, and amino acids were then assessed using liquid chromatography with tandem mass spectrometry (LC-MS; LC-MS8060; Shimadzu Corporation, Kyoto, Japan).

### Measurement of mitochondrial hydrogen peroxide in vivo

We prepared the MitoB (C_25_H_23_BBrO_2_P, molecular weight 477.14; Sigma–Aldrich Japan K.K., Tokyo, Japan) stock solution in sterile saline. Under general anaesthesia, laminectomy was performed at the fifth lumbar vertebrae in 8-week-old ICR mice. The catheter was filled with the diluted MitoB solution (0.5 mg/ml in a 1:1 solution of DMSO: PBS). Holding the fourth lumbar spinous process, we inserted the catheter (0.8 Fr/32Ga/PU/14 cm, Insitech Laboratories, Inc., Plymouth Meeting, PA, USA) into the subarachnoid space. After the 10th thoracic laminectomy, we confirmed the presence of the catheter in the subarachnoid space. The catheter was placed just caudal to the injury. Twenty microlitres of the diluted MitoB solution was injected using a syringe. SCI was induced using a disposable straight clip (AM-1; Natsume Seisakusho, Tokyo, Japan) for 15 s. Twenty minutes (*n* = 3) and 6 h (*n* = 5) after decompression, we euthanized the mice, dissected the spinal cords with bones, and flash-froze the spinal cord in liquid nitrogen stored at −80°C. The spinal cord was dissected into rostral, epicentre, and caudal segments, each of which was 2 mm in length. The production of mitochondrial hydrogen peroxide was assessed as previously described.^13^ Hydrogen peroxide was then assessed by the determination of the MitoP/MitoB ratio using LC-MS (LC-MS8040; Shimadzu Corporation).^14^

### Mitochondrial superoxide detection

Six hours after SCI, T9–T12 laminectomy was performed with 30 g/mm^2^ of clipping power at a duration of 15 s (*n* = 5). The dura mater was gently peeled off, thereby exposing the pia of the spinal cord. Then, 5 μM of MitoSox (Thermo Fisher Scientific) was used to cover the spinal cord. Then, the spinal cord was incubated for 10 min under anaesthesia and was protected from light. Next, it was washed twice with PBS, and assessments were performed with a Leica M165 FC stereo microscope (Leica) using a 2× objective (Leica, Plan Apo 1.0) with a 510/580 emission/excitation filter with an exposure time of 2 s. Three ROIs (0.5 mm in diameter, round circle) were set at the epicentre and rostro-caudal segment, 3 mm from the epicentre. The imaging data were analysed using MetaMorph (Molecular Devices). The signal intensity at the chosen ROI was subtracted from the background intensity without the application of MitoSox.

### A lipid peroxidation analysis

Six (*n* = 5) and 24 h (*n* = 6) after SCI with 30 g/mm^2^ of clipping power for a duration of 15 s, the spinal cord was dissected into rostral, epicentre, and caudal segments, each of which was 2 mm in length. Each spinal cord segment was minced with a homogenizer in RIPA buffer containing 50-mM Tris–HCL 7.5 (Nippon Gene Co., Ltd), 150-mM NaCl (Nacalai Tesque), 0.1% SDS (Nippon Gene Co., Ltd), 1% NP-40 (Sigma-Aldrich), 5-mM EDTA with pH 8.0 (Nippon Gene Co., Ltd ), and 1 mM PMSF (Wako). Protease inhibitor cocktail (Thermo Fisher Scientific) was added to the buffer prior to homogenization in accordance with the manufacturer’s instructions (1:100). The spinal cord was homogenized for ∼40 grinds using constant force to ensure consistency and homogeneity of the samples. The homogenates were divided into roughly equal volumes in Eppendorf tubes and stored at 4°C for 30 min. The homogenates were then centrifuged at 13 000 rpm for 20 min at 4°C. The resulting supernatant was decanted, placed into newly labelled Eppendorf tubes, and immediately stored at −80°C. The protein concentration was determined using Qubit (Thermo Fisher Scientific). Equal amounts of protein were examined. Lipid peroxidation was quantified as a marker of oxidative stress using the OxiSelect 4-HNE Adduct Competitive ELISA Kit (Cell Biolabs, Inc., San Diego, CA, USA).

### Statistical analyses

All statistical analyses were performed using the XLSTAT software program (Paris, France). Wilcoxon’s signed-rank test was used to compare continuous variables between two groups. For comparisons among multiple groups, data were analysed using the Kruskal–Wallis test along with Dunn’s post hoc test. The values were presented as the mean±SD.

### Data availability

Study data are available in Supplementary Data Set.

## Results

### Thoracic compression injury model

**Figure 1:**
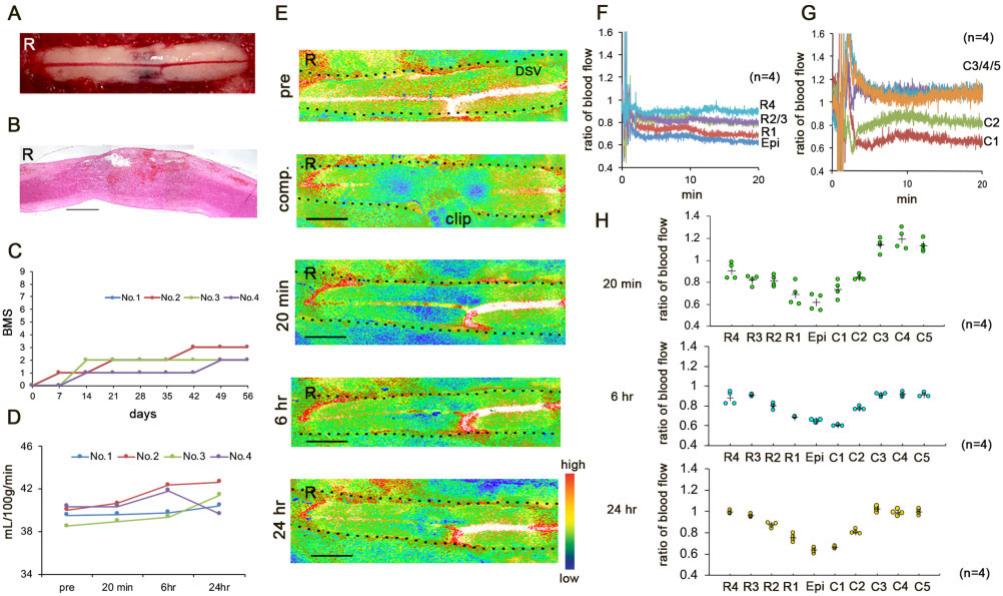
**(A–C) The thoracic compression injury model.** (**A**) Representative image obtained 20 min after decompression. (**B**) Haematoxylin and eosin staining in the mid-sagittal section 24 h after SCI. R, rostral. Scale bar = 1 mm. (**C**) Basso Mouse Scale scores up to 8 weeks after SCI. Error bars represent the mean±SD. (**D–H**) Real-time blood flow distributions in early SCI. (**D**) Blood flow values at 5-mm rostral from the epicentre before SCI (pre-SCI) and 20 min, 6 h, and 24 h after SCI. Error bars represent the mean±SD. (**E**) Representative speckle contrast images of the spinal cord obtained before SCI (pre-SCI), during compression, and 20 min, 6 h, and 24 h after SCI. The dotted lines indicate the border of the spinal cord. R, rostral; DSV, dorsal spinal vein. Scale bar = 2 mm. (**F** and **G**) The mean ratio of the blood flow at the rostral/caudal points from the epicentre 20 min after SCI. (**H**) The ratio of the blood flow 20 min, 6 h and 24 h after SCI. R/C1, 2, 3, 4, and 5 are located 1, 2, 3, 4, and 5 mm rostral and caudal from the epicentre, respectively. Data were analysed using the Kruskal–Wallis test along with Dunn’s post hoc test. Plus marks represent the mean. (*n* = 4).

The clip that was used to create the SCI compresses the spinal cord at a specific force and duration and occludes the blood supply in a specific cord segment.^15^ The haemorrhagic lesion extended rostro-caudally from the epicentre of injury (Fig. 1A). The dorsal portion, including the most ventral part of the dorsal funiculus, which includes the corticospinal tract, was damaged (Fig. 1B).^16^ The BMS score was 0 at 24 h after SCI and 2.33 ± 0.57 [mean±SD] at 8 weeks after SCI, indicating severe locomotor impairment (Fig. 1C).

### Real-time blood flow distributions in early SCI

To study the spatiotemporal blood supply in SCI, the blood flow was monitored in real-time with LSF.^17^^,^^18^ LSF detects the scattered light of red blood cells to measure the blood flow (ml) per minute in 100 g and provides relative values for the blood flow. The average blood flow values at R5 (5 mm rostral from the epicentre) were stable at 39.89 ± 0.76 (mean±SD) ml/min/100 g (Fig. 1D). There were no significant differences in the blood flow values at R5 at 20 min, 6 h, and 24 h after SCI. We evaluated the changes in blood distribution according to the ratio of the blood flow at R5.

During compression, the hypoperfused region extended rostro-caudally (Fig. 1E). At 20 min after SCI, the average blood flow ratio at the epicentre was 0.61 ± 0.17. We considered that a longer duration of compression would cause greater extension of the low blood flow in the rostro-caudal areas due to the longer cessation of the blood supply. However, the blood flow was significantly increased at C3/4/5 (3/4/5 mm caudal from the epicentre, respectively) (*P* = 0.002) between 20 min and 6 h after SCI (*P* = 0.002, *P* = 0.001, *P* = 0.003) (Fig. 1G and H). The blood flow at R3 was significantly re-perfused between 20 min and 24 h after SCI (*P* = 0.002) (Fig. 1H). These findings indicated reperfusion on the rostral side of the injury and stable perfusion on the caudal side.

### Rostro-caudal differences in ATP levels in early SCI

**Figure 2:**
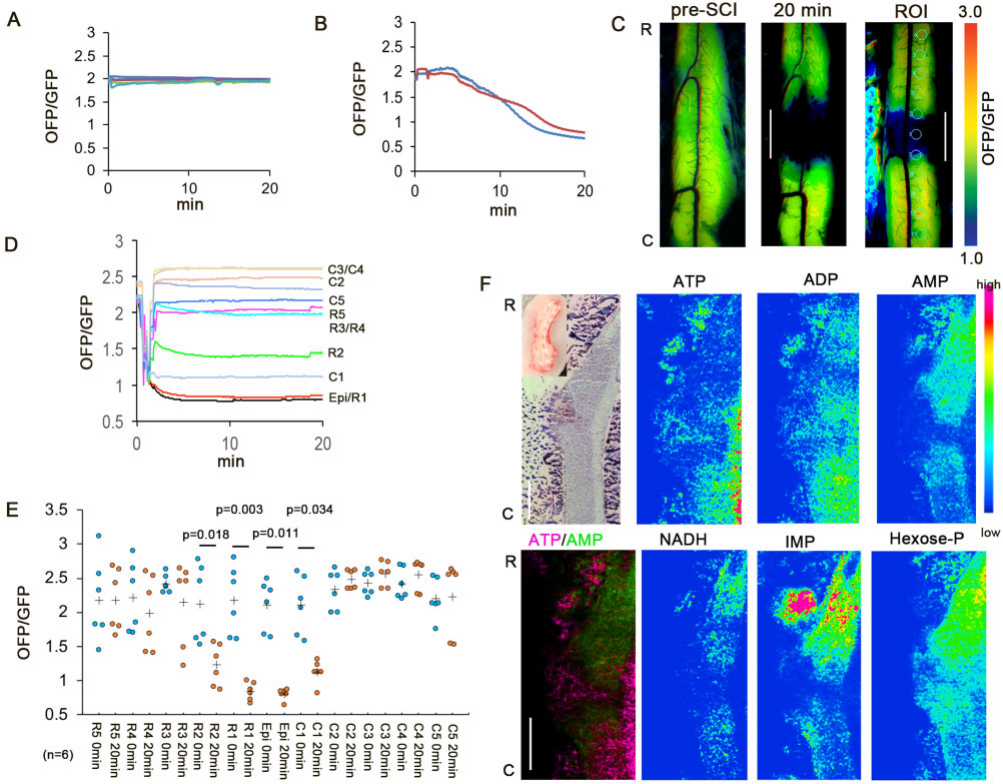
**Real-time monitoring of intracellular ATP.** (**A**) Colour traces represent the OFP/GFP ratio in the normal spinal cord (*n* = 5). (**B**) Red and blue traces represent the OFP/GFP ratio in the normal spinal cord after cardiac circulation arrest (*n* = 2). (**C**) Representative intracellular ATP images at pre-SCI and 20 min after SCI. The maximum OFP/GFP value at 3.0 and the minimum value at 1.0. The right panel shows the ROI. Scale bar = 2 mm. (**D**) Intracellular ATP reduction on the rostral side of the injury. Colour traces represent the mean OFP/GFP ratio after decompression (*n* = 6). (**E**) Intracellular ATP level before SCI (pre-SCI) and 20 min after SCI. Plus marks represent the mean. (*n* = 6). (**F**) MS findings at 20 min after SCI (*n* = 4). The caudal side had a high ATP level. The NADH, hexose phosphate, and IMP levels were high on the rostral side. 2,3-Diphosphoglyceric acid (2,3-DPG) was observed at the injury site. NADH, nicotinamide adenine dinucleotide; IMP, inosine5′-monophosphate; Hexose-P, hexose phosphate; R, rostral. C, caudal. Data were analysed using Wilcoxon’s signed-rank test and the Kruskal–Wallis test along with Dunn’s post hoc test. Scale bar = 1 mm.

To clarify the state of cellular energy in early SCI, we examined the intracellular ATP using GO-ATeam2 Tg mice.^19^^,^^20^ The average OFP/GFP ratio was 1.98 ± 0.14 in the normal spinal cord (Fig. 2A), and the average ATP value was 2.02 mM. This value matched the previous reported data, which showed that the physiological intracellular concentration was ∼1–5 mM.^21^^,^^22^ At 20 min after circulation arrest, the average OFP/GFP ratio was 0.71 ± 0.08 (Fig. 2B), and the average ATP value was 0.09 mM. This ATP level indicates severe cellular degeneration. The intracellular ATP levels were decreased at R1/C1 (Fig. 2C and D). The reduction in the ATP level expanded rostrally, while the caudal side of the injury showed preserved ATP levels (Fig. 2D and E).

To spatially explore the metabolic alterations in SCI, we performed MS imaging of spinal cords at 20 min after SCI. Consistent with the above results, the tissue ATP levels were higher on the caudal side of the injury than the rostral side (Fig. 2F and Supplementary Fig. 1). A coronal view also revealed that the tissue ATP levels were lower in the grey matter at the rostral side of the injury than the white matter (Supplementary Fig. 1). Notably, cells on the rostral side accumulated NADH, which indicated the disruption of the acute mitochondrial electron transport chain (ETC) activity as well as AMP and IMP, reflecting ATP consumption and break down, respectively.^23^^,^^24^ The hexose phosphate levels were higher on the caudal side of the injury than the rostral side. The injury site had increased levels of 2,3-bisphosphoglycerate, an indicator of erythrocytes, and decreased levels of lysophosphatidic acid. These results suggest that the rostral side had an ATP shortage due to a reduced production of ATP as a result of ETC disruption as well as elevated ATPase activity; in contrast, the ATP levels on the caudal side were maintained at 20 min after SCI.

### Rostro-caudal differences in energy metabolites in early SCI

**Figure 3:**
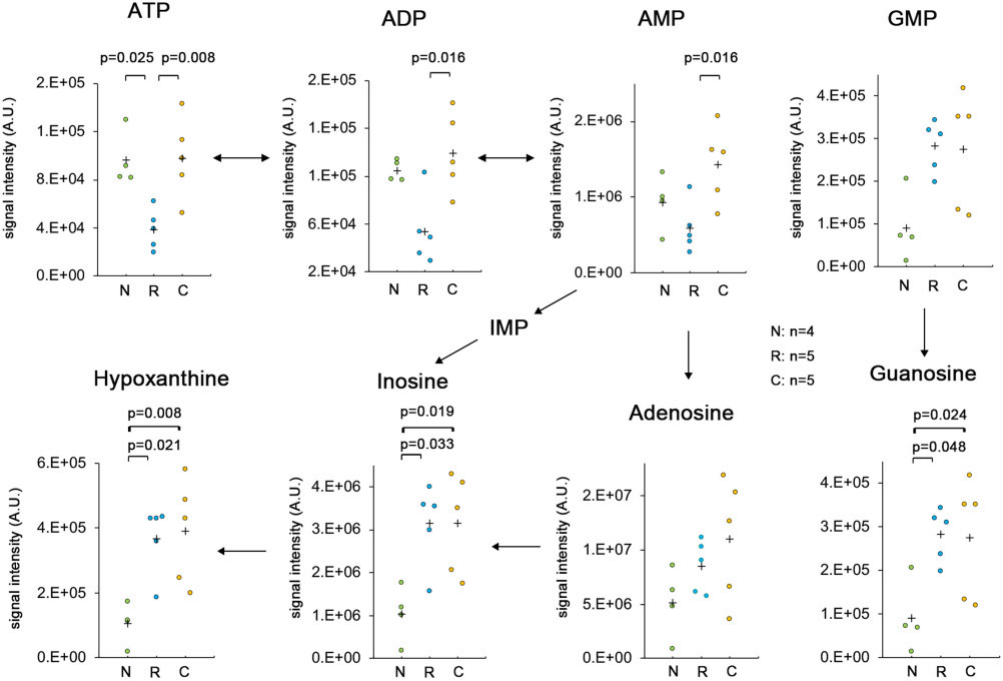
**The breakdown products at the rostral and caudal sides of injury and in the sham-operated spinal cord.** A metabolome analysis was conducted for the spinal cord segment at the rostral and caudal sides of injury and in the sham-operated spinal cord at 18 h after SCI. The contents of purine nucleotides (ATP, ADP, AMP, GMP) and breakdown products (Adenosine, Guanine, Inosine, Hypoxanthine) were estimated. N, sham-operated spinal cord (*n* = 4); R, rostral side of injury (*n* = 5); C, caudal side of injury (*n* = 5). Data were analysed using Wilcoxon’s signed-rank test and the Kruskal–Wallis test along with Dunn’s post hoc test. Plus marks represent the mean.

To determine the rostro-caudal energy metabolism in early SCI, we conducted metabolome analyses for the spinal cord segment at the rostral and caudal sides of injury as well as in sham-operated cases at 18 h after SCI. The levels of purine nucleotides (ATP, ADP, AMP, GMP) and breakdown products (adenosine, guanosine, inosine, hypoxanthine) were estimated. At the caudal side of the injury, the ATP level was the same as in the sham-operated spinal cord and was significantly higher than that at the rostral side (Fig. 3). However, there was no rostro-caudal difference in signal intensity for hypoxanthine, guanine, or inosine (Fig. 3). These breakdown products at the rostral and caudal side were significantly elevated compared to the sham-operated spinal cord. While adenosine is a breakdown product of ATP that is increased in ischaemic regions, there was no significant accumulation at the rostral side of injury. The levels of other degradative products of ATP (i.e. ADP and AMP) were significantly different between the rostral and caudal sides of injury. These results suggest that tissue degradation occurred at both sides of the injury, but ATP production was maintained on the caudal side.

**Figure 4:**
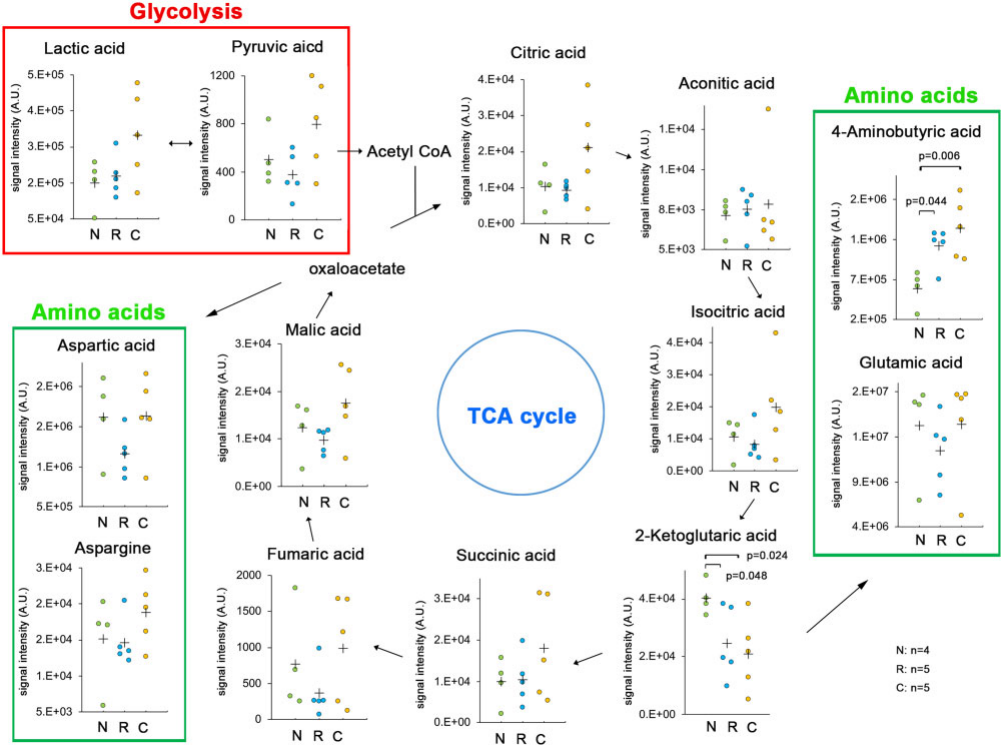
**Principal metabolites in the glycolytic pathway, TCA cycle, and amino acids at the rostral and caudal sides of injury and in the sham-operated spinal cord.** A metabolome analysis was conducted for the spinal cord segment at the rostral and caudal sides of injury and in the sham-operated spinal cord at 18 h after SCI. N, sham-operated spinal cord (*n* = 4); R, rostral side of injury (*n* = 5); C, caudal side of injury (*n* = 5). Data were analysed using Wilcoxon’s signed-rank test and the Kruskal–Wallis test along with Dunn’s post hoc test. Plus marks represent the mean.

To examine the energy maintenance at the caudal side of injury, the levels of principal metabolites in the glycolytic pathway, TCA cycle, and amino acids were estimated at the rostral and caudal sides of injury as well as in the sham-operated spinal cord at 18 h after SCI (Fig. 4). Pyruvic acid and citric acid were accumulated at the caudal side, but not to a significant degree. The levels of lactic acid from the glycolytic pathway were higher at the caudal side than at the rostral side of injury or in the sham-operated spinal cord. Although the levels of 2-ketoglutaric acid were significantly decreased on the caudal side of injury, the production of 4-aminobutyric acid was preserved. These findings suggest that the caudal side maintained ATP production through the glycolytic pathway and TCA cycle.

### Rostro-caudal differences in degeneration in early SCI

**Figure 5:**
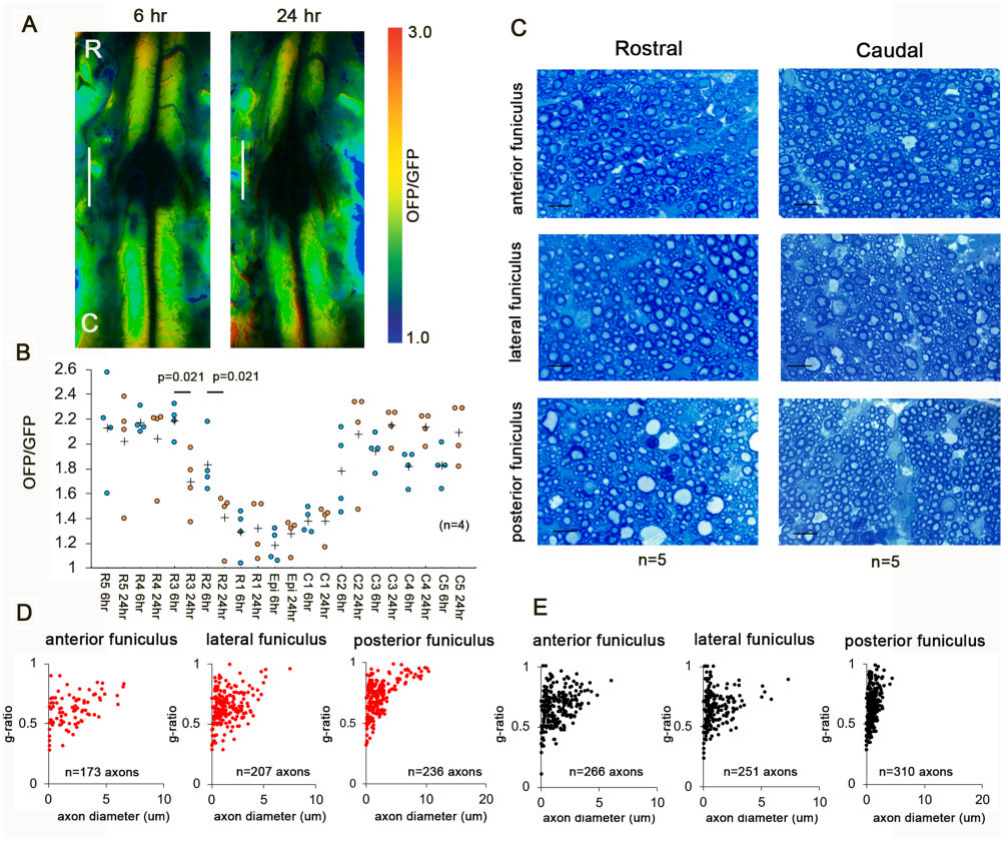
**Rostro-caudal differences in degeneration in early SCI.** (**A**) Representative intracellular ATP images at 6 h and 24 h after decompression. Scale bar = 2 mm. (**B**) Intracellular ATP levels at 6 and 24 h after SCI. (*n* = 4). Caudal degeneration in early SCI. Plus marks represent the mean. (**C**) Representative images in white matter cross-sections 24 h after SCI at 3 mm rostral and caudal from the epicentre. Scale bar = 10 um. (**D** and **E**) Scatterplot of the g-ratio versus axon diameter in white matter cross-sections at 3 mm rostral (**D**) and caudal (**E**) from the epicentre. (*n* = 5). Data were analysed using Wilcoxon’s signed-rank test and the Kruskal–Wallis test along with Dunn’s post hoc test.

To identify the changes in cellular energy in early SCI, we studied the intracellular ATP levels at 6 and 24 h after SCI. The low ATP levels expanded to the rostral side of the injury (Fig. 5A and B), while the ATP level on the caudal side of the injury did not change. We examined the blood flow distribution and ATP levels in the shorter-duration (3 s) compression models with the same severe locomotor deficits. The rostro-caudal blood flow distribution and ATP levels were preserved until 24 h after SCI (Supplementary Fig. 2). To clarify the degree of degeneration in the white matter, we investigated the axonal morphology. Representative images in white matter cross-sections 24 h after SCI at 3 mm rostral and caudal from the epicentre were obtained (Fig. 5C). Figure 5D and E shows a scatterplot of the g-ratio versus the axon diameter in white matter cross-sections at 3 mm rostral and caudal from the epicentre. The axon diameter at the rostral side was significantly enlarged in the anterior, lateral, and posterior funiculus (Table 1). In contrast, the g-ratio at the rostral side indicated significant thinning of the myelin sheath in the posterior funiculus (Table 1).

**Table 1:** Axon diameter and g-ratio in white matter cross-sections at 3 mm rostral and caudal from the epicentre

	White matter	Axon diameter (μm)		*P* value	g-Ratio		*P* value
		Rostral	Caudal		Rostral	Caudal	
SCI	Anterior funiculus	2.01 ± 1.56	1.36 ± 1.10	<0.0001	0.61 ± 0.13	0.65 ± 0.15	0.015
	Lateral funiculus	1.55 ± 1.26	0.99 ± 1.06	<0.0001	0.63 ± 0.13	0.65 ± 0.14	0.068
	Posterior funiculus	2.21 ± 2.18	1.18 ± 0.68	<0.0001	0.69 ± 0.14	0.63 ± 0.13	<0.0001
2-DG	Anterior funiculus	1.89 ± 1.19	2.04 ± 1.72	0.441	0.61 ± 0.11	0.61 ± 0.11	0.716
	Lateral funiculus	2.32 ± 2.03	3.44 ± 3.21	0.001	0.61 ± 0.12	0.67 ± 0.14	0.0005
	Posterior funiculus	2.45 ± 2.18	3.02 ± 3.15	0.082	0.62 ± 0.11	0.66 ± 0.12	0.006

SCI = spinal cord injury; 2DG = 2DG treated SCI.

### Rostro-caudal different degeneration after 2-DG treatment

**Figure 6:**
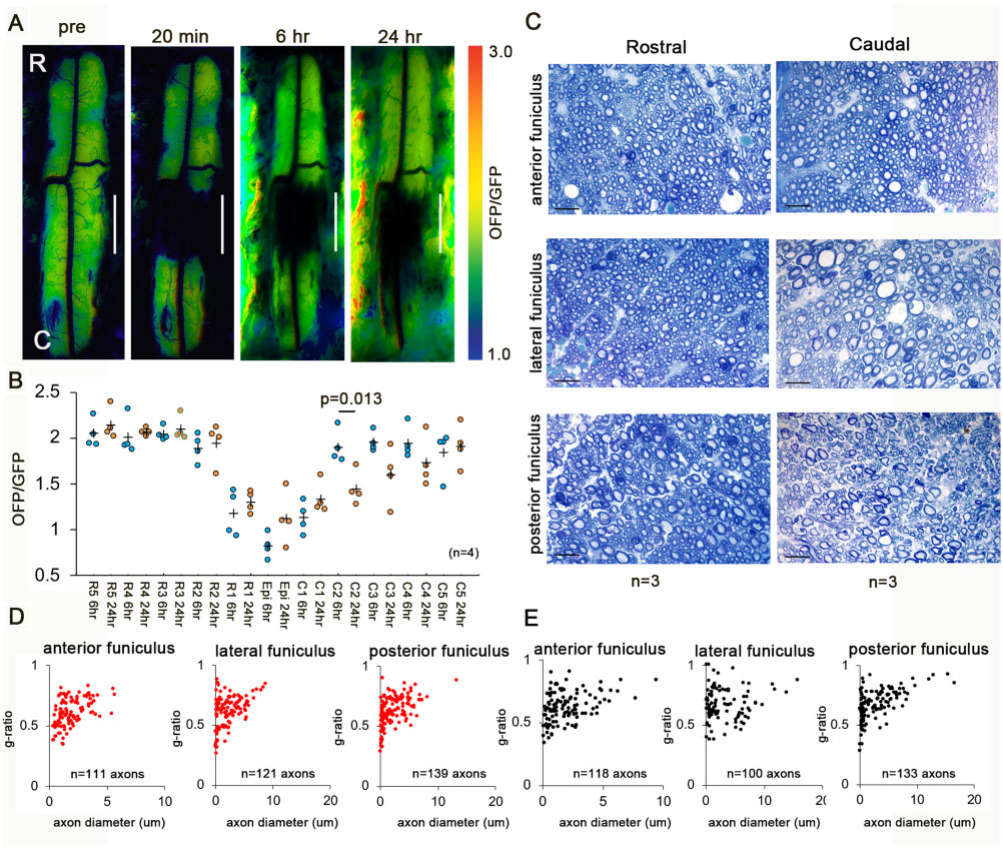
**Rostro-caudal differences in degeneration in early SCI after 2-DG treatment.** (**A**) Representative intracellular ATP images before and 20 min, 6 h, and 24 h after SCI with 2-DG administration. (**B**) The intracellular ATP levels at 6 and 24 h after SCI. The maximum OFP/GFP value at 3.0 and the minimum value at 1.0. Data were analysed using Wilcoxon’s signed-rank test and the Kruskal–Wallis test along with Dunn’s post hoc test. Plus marks represent the mean. (*n* = 4). (**C**) Representative images in white matter cross-sections 24 h after SCI at 3 mm rostral and caudal from the epicentre. Scale bar = 10 um. (**D** and **E**) Scatterplot of g-ratio versus axon diameter in white matter cross-sections at 3 mm rostral (**D**) and caudal (**E**) from the epicentre. (*n* = 3). Data were analysed using Wilcoxon’s signed-rank test and the Kruskal–Wallis test along with Dunn’s post hoc test.

Next, we checked the caudal energy metabolism. 2-DG blocks glycolysis, thereby reducing cellular ATP. The administration of 2-DG inverted the asymmetry of the rostro-caudal ATP levels (Fig. 6A and B). The ATP levels were decreased on the caudal side but did not change on the rostral side. These results suggest that caudal degeneration is involved in ATP production via the glycolytic pathway in early SCI, indicating that mitochondrial ATP was insufficient to support the caudal energy process. To clarify the degeneration in the white matter, we checked the axonal morphology. Representative images in white matter cross-sections 24 h after SCI at 3 mm rostral and caudal from the epicentre were obtained (Fig. 6C). Figure 6D and E show a scatterplot of the g-ratio versus the axon diameter in white matter cross-sections at 3 mm rostral and caudal from the epicentre. The axon diameter at the caudal side was significantly enlarged in the lateral funiculus but not in the anterior or posterior funiculus (Table 1). In contrast, the g-ratio at the caudal side indicated significant thinning of the myelin sheath in the lateral and posterior funiculus (Table 1).

### Rostro-caudal differences in oxidative stress in early SCI

**Figure 7:**
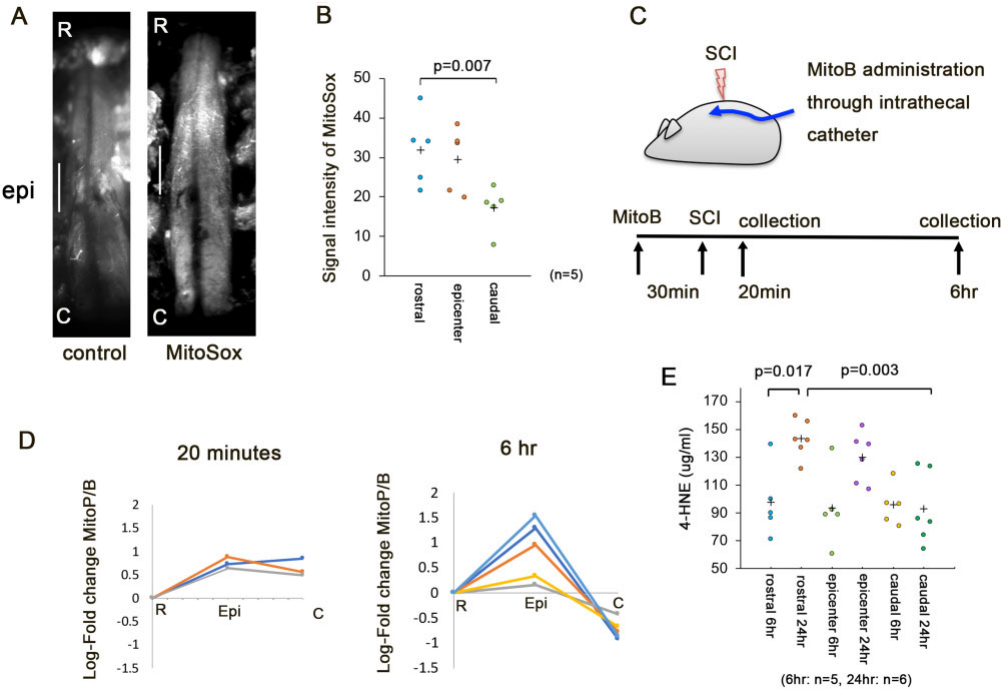
**Rostral oxidative stress in early SCI.** (**A**) Left, control view; right, representative view with MitoSox. Scale bar = 2 mm. (**B**) Signal intensity of MitoSox in the rostral, epicentre, and caudal segments at 6 h after decompression. (**C**) A schematic illustration of the administration of MitoB. The intrathecal catheter was inserted into the subarachnoid space at the fifth lumbar vertebra. At 30 min after the injection of MitoB from the catheter, SCI was induced. The spinal cord was dissected into rostral, epicentre, and caudal segments. (**D**) The log-fold change in the MitoP/MitoB ratio in the rostral, epicentre, and caudal segments at 20 min (*n* = 3) and 6 h (*n* = 5) after SCI. Hydrogen peroxide levels was then assessed by determination of the MitoP/MitoB ratio using LC–MS/MS (LC-MS8040, Shimadzu Corporation). (**E**) The 4-HNE level was quantified in the rostral, epicentre, and caudal segments in the 15-s group at 6 h (*n* = 5) and 24 h after decompression (*n* = 6). Data were analysed using the Kruskal–Wallis test along with Dunn’s post-test. Plus marks represent the mean.

To clarify the rostro-caudal difference in oxidative stress, we studied the mitochondrial ROS production in early SCI. The MitoSox probe rapidly accumulates in mitochondria and detects superoxide production within mitochondria.^25^ The superoxide level was significantly higher at the rostral side of injury than at the caudal side at 6 h after SCI (Fig. 7A and B). Next, we investigated the production of hydrogen peroxide in the rostral, epicentre, and caudal segments at 2-mm intervals (Fig. 7C). MitoB accumulates rapidly within mitochondria and should be converted to MitoP in proportion to the local hydrogen peroxide concentration.^26^ Although the MitoP/B ratios at the epicentre and caudal segment were higher than those in the rostral segment at 20 min after decompression, the MitoP/B ratio in the caudal segment was lower than that in the rostral segment at 6 h after decompression (Fig. 7D). To examine the lipid peroxidation caused by cellular oxidative stress, we also assessed the 4-hydroxynonenal levels at 6 and 24 h after decompression. The 4-hydroxynonenal levels in the rostral, epicentre, and caudal segments did not differ significantly at 6 h after SCI. However, the level in the rostral segment was significantly higher than that in the caudal segment at 24 h after decompression (Fig. 7E). These results suggest that rostral oxidative stress exacerbated the degeneration in early SCI.

## Discussion

In this study, real-time monitoring of intracellular ATP showed that the rostral side of the injury had an ATP shortage due to reduced production, while the ATP levels were maintained on the caudal side. Breakdown products of purine nucleotides were accumulated at both sides of injury. The principal metabolites in the TCA cycle and glycolytic pathway were elevated on the caudal side. Although the low-ATP regions expanded to the rostral side of injury until 24 h after SCI, the ATP levels on the caudal side were preserved. The administration of 2-DG decreased the ATP levels on the caudal side but did not change them on the rostral side.

The compression model occludes arterial blood flow in a specific cord segment and inhibits venous return. The thoracic segmental arteries of adult mice are derived from the ventral spinal artery.^27–29^ The segmental artery follows a course from the rostral side at a regular angle. Clip compression seems to inhibit the rostral vascular supply in the thoracic cord. Rostral reperfusion is considered to cause oxidative stress. However, the imbalance in the blood flow distribution after compression injury seems to depend on the territory of the segmental artery. The central nervous system has control systems that ensure an adequate blood supply, including the dilation and constriction of blood vessels. The caudal vessels in SCI lose monoamine innervation, which arises from brainstem neurons.^30^ The loss of monoamine leads to the dilation of blood vessels in SCI. Therefore, stable perfusion on the caudal side of the injury may be due to the loss of monoamine and venous congestion.

In the current study, the axon diameter at the rostral side was significantly enlarged in the anterior, lateral, and posterior funiculus. The oxidative stress induced by rostral reperfusion injury was considered to have caused axon degeneration independent of the ascending and descending tracts. In contrast, the myelin sheath in the posterior funiculus showed significant thinning, suggesting severe damage to the posterior funiculus at the rostral side, because the dorsal and lateral portions were damaged (Fig. 1B and Supplementary Fig. 1). Interestingly, following 2-DG treatment, the axon diameter at the causal side was significantly enlarged in the lateral funiculus. The descending tracts in the lateral funiculus transport mitochondria to the caudal side, therefore it may be more dependent on the glycolytic energy in SCI.

Mitochondrial ROS not only drive acute damage but also initiate multiple cascades of pathological entities that develop over several weeks following reperfusion.^31–33^ The initial burst of ROS production directly causes oxidative damage to the mitochondria, thereby disrupting ATP production. The induction of cellular oxidative stress initiates the peroxidation and destruction of lipids. In the current study, the superoxide and hydrogen peroxide levels in the rostral region significantly increased prior to the reduction of intracellular ATP. Furthermore, after transection of the spinal cord in adult mice, the severed axons several hundred micrometres from the injury site die within 30 min of injury and then promptly start regenerating.^1^ Thus, the rostral severed axons were likely damaged by oxidative stress in early SCI.

Disruption of axonal transport leads to a disorganized distribution of mitochondria. Sufficient levels of ATP can be generated in the absence of mitochondria to support active axonal processes.^34^^,^^35^ Localized glycolytic machinery may supply constant energy, independent of mitochondria, for the processive movement of vesicles over long distances in axons. In the present study, the administration of 2-DG decreased ATP levels on the caudal side but did not change the levels on the rostral side. 2-DG induces endoplasmic reticulum stress, leading to the activation of autophagy.^34^ Mitophagy attenuated mitochondrial dysfunction and ROS generation.^36^^,^^37^ 2-DG not only blocks glycolysis, thereby reducing cellular ATP, but it may also alleviate mitochondrial ROS production in early SCI.

ROS is an important contributor to secondary damages of SCI. ROS-mediated mitochondrial damage releases damage-associated molecular pattern molecules and can cause a continuous signalling cascade of secondary events.^38^ There are a number of non-mitochondrial ROS sources, which include NADPH oxidases,^39^ xanthine oxidases,^40^ and uncoupled NO synthase^41^ Although non-mitochondrial ROS are important contributors to the secondary progression of the pathology, activation of these processes is thought to occur after the initial burst of mitochondrial ROS.^32^^,^^42^^,^^43^ The initial burst of ROS production directly causes oxidative damage to mitochondria, thereby disrupting ATP production.^41^ The elevated ROS production also leads to mitochondrial permeability transition with dysregulation of the calcium levels, followed by cell death.

In the present study, the superoxide and hydrogen peroxide levels in the rostral region were significantly increased prior to the reduction in intracellular ATP levels. Furthermore, in the lesion, the superoxide level was higher at 6 h after SCI than the caudal side of injury (Fig. 7B). The MitoP/B ratio at the injury segment was also higher at 6 h after SCI than the rostral or caudal side of injury (Fig. 7D), and the level of 4-hydroxynonenal in the injury segment was higher at 24 h after SCI than 6 h after SCI (Fig. 7E). These results suggest that oxidative stress exacerbated secondary injury of the injured segment.

Thoracic compression injury models with three different durations of compression presented with differing histological severities with the same severe locomotor deficits (data not shown). The BMS scores were 3.40 ± 0.89, 3.52 ± 1.70, and 2.33 ± 0.57 in the 3-, 7-, and 15-s groups, respectively, at 8 weeks after SCI and not significantly different among the groups. The epicentre areas were significantly different between the 3- and 15-s groups. A shorter compression mitigated tissue damage. Longer compression showed the reperfusion area in the rostral side of the injury. Such regional reperfusion induces the mitochondrial metabolism to produce excessive ROS. The real-time fluorescence resonance energy transfer-based monitoring of intracellular ATP visualized that regional ATP drop down in rostral reperfusion areas. Early decompression in severe SCI results in an imbalance in the blood flow distribution followed by asymmetric lesion expansion initiated by a burst of mitochondrial ROS, suggesting a counteracting effect to reduce histological damage. Given these findings, we propose treatment be initiated to protect mitochondria and maintain the blood flow in SCI patients in order to alleviate damage to the lesion.

## Conclusions

In this study, we showed that rostro-caudal differences in energy metabolism led to differences in the spread of degeneration in early thoracic SCI. Our results suggest that the glycolytic pathway is involved in the delayed caudal degeneration, while immediate rostral degeneration is exacerbated by oxidative stress in early thoracic SCI.

## Supplementary material

Supplementary material is available at *Brain Communications* online.

## Supplementary Material

fcab058_Supplementary_Data

## Abbreviations

2-DG: 2-deoxy-d-glucose
GFP: green fluorescent protein
LSF: laser speckle flowgraphy
OFP: orange fluorescent protein
ROS: reactive oxygen species
SCI: spinal cord injury
