# Supplementary material for: Rostro-caudal different energy metabolism leading to differences in degeneration in spinal cord injury
Source: Brain Commun. 2021 Mar 28;3(2):fcab058. doi: 10.1093/braincomms/fcab058 (PMC8066884; doi:10.1093/braincomms/fcab058)
Supplement: fcab058_Supplementary_Data [file fcab058_supplementary_data.zip › Data Fig1C,D,H; Fig2E; Fig5B; Fig6B; Fig7B,E.docx]

**Figure 1C**

|  | Day 0 | Day 7 | Day 14 | Day 21 | Day 28 | Day 35 | Day 42 | Day 49 | Day 56 |
| --- | --- | --- | --- | --- | --- | --- | --- | --- | --- |
| 1 | 0 | 1 | 1 | 2 | 2 | 2 | 3 | 3 | 3 |
| 2 | 0 | 1 | 1 | 2 | 2 | 2 | 3 | 3 | 3 |
| 3 | 0 | 0 | 2 | 2 | 2 | 2 | 2 | 2 | 2 |
| 4 | 0 | 0 | 1 | 1 | 1 | 1 | 1 | 2 | 2 |

**Figure 1D**

|  | pre (mL/100g/min) | 20 min (mL/100g/min) | 6 hr (mL/100g/min) | 24 hr (mL/100g/min) |
| --- | --- | --- | --- | --- |
| 1 | 39.52 | 39.63 | 39.79 | 40.45 |
| 2 | 40.01 | 40.65 | 42.36 | 42.67 |
| 3 | 38.51 | 38.95 | 39.38 | 41.41 |
| 4 | 40.37 | 40.37 | 41.83 | 39.64 |

**Figure 1H**

**20min**

| Epi | R1 | R2 | R3 | R4 | C1 | C2 | C3 | C4 | C5 |
| --- | --- | --- | --- | --- | --- | --- | --- | --- | --- |
| 0.547304812 | 0.619017335 | 0.759898236 | 0.826681517 | 0.950079257 | 0.715492561 | 0.882207485 | 1.162761716 | 1.109785823 | 1.131635066 |
| 0.558056299 | 0.60494458 | 0.78312023 | 0.852003422 | 0.840843752 | 0.768635957 | 0.826045636 | 1.052231995 | 1.303806938 | 1.084877001 |
| 0.67690958 | 0.706935925 | 0.839859571 | 0.756628679 | 0.987532929 | 0.826045636 | 0.838779653 | 1.139644513 | 1.127059621 | 1.210782867 |
| 0.688079267 | 0.824544619 | 0.875359538 | 0.844359261 | 0.840843752 | 0.638209072 | 0.829965623 | 1.206880411 | 1.239806911 | 1.101189917 |

**6Hr**

| Epi | R1 | R2 | R3 | R4 | C1 | C2 | C3 | C4 | C5 |
| --- | --- | --- | --- | --- | --- | --- | --- | --- | --- |
| 0.630154634 | 0.692493545 | 0.765141725 | 0.919458794 | 0.830101502 | 0.612859387 | 0.770904266 | 0.930590527 | 0.90839129 | 0.925114758 |
| 0.662792619 | 0.694006321 | 0.807903054 | 0.922795928 | 0.824447731 | 0.600817637 | 0.766260118 | 0.915214817 | 0.889917248 | 0.898809519 |
| 0.644950534 | 0.695365483 | 0.834212474 | 0.898455142 | 0.932126924 | 0.601097157 | 0.803870234 | 0.893932803 | 0.924814493 | 0.902371556 |
| 0.662752307 | 0.687593685 | 0.815592212 | 0.914730154 | 0.950742561 | 0.6087782 | 0.775931092 | 0.927670042 | 0.949445966 | 0.944444459 |

**24Hr**

| Epi | R1 | R2 | R3 | R4 | C1 | C2 | C3 | C4 | C5 |
| --- | --- | --- | --- | --- | --- | --- | --- | --- | --- |
| 0.666247651 | 0.801379445 | 0.894453172 | 0.954358184 | 1.017281363 | 0.66453433 | 0.801173477 | 1.052959976 | 1.022229709 | 1.028589106 |
| 0.650819715 | 0.768108182 | 0.889806737 | 0.96399624 | 0.980995718 | 0.663036647 | 0.79596201 | 0.99257758 | 0.955907932 | 0.990738959 |
| 0.60879618 | 0.71666342 | 0.840964929 | 0.946527333 | 0.985650565 | 0.676859124 | 0.839803761 | 1.037864552 | 0.986734654 | 1.015049112 |
| 0.62538187 | 0.739378668 | 0.870445927 | 0.983197223 | 0.992704967 | 0.6501014 | 0.805289039 | 1.018442874 | 0.981955567 | 0.960139401 |

**Figure 2E**

| R5 0 | 3.114172 | 1.821195 | 2.323486 | 1.810588 | 2.560832 | 1.43975 |
| --- | --- | --- | --- | --- | --- | --- |
| R5 20 | 1.816835 | 1.799286 | 2.431087 | 1.66 | 2.683318 | 2.622593 |
| R4 0 | 2.899946 | 1.708119 | 2.459396 | 1.699667 | 2.647386 | 1.843005 |
| R4 20 | 1.681554 | 1.420419 | 2.531817 | 1.4 | 2.284181 | 2.604142 |
| R3 0 | 2.291156 | 2.624861 | 2.288504 | 2.369531 | 2.522421 | 2.383007 |
| R3 20 | 1.488087 | 1.21234 | 2.456939 | 2.457489 | 2.578596 | 2.646833 |
| R2 0 | 2.774626 | 1.673453 | 2.457829 | 1.524287 | 2.641952 | 1.616832 |
| R2 20 | 0.87311 | 0.905762 | 1.35688 | 1.110172 | 1.572021 | 1.523719 |
| R1 0 | 2.804913 | 1.979559 | 2.436174 | 1.625486 | 2.579373 | 1.61128 |
| R1 20 | 0.6645 | 0.962888 | 0.807509 | 0.725613 | 0.867633 | 1.005095 |
| Epi 0 | 2.320849 | 2.148928 | 2.372114 | 1.631503 | 2.495368 | 1.672519 |
| Epi 20 | 0.632365 | 0.844066 | 0.868717 | 0.793836 | 0.841546 | 0.803048 |
| C1 0 | 2.063642 | 2.518689 | 2.192154 | 2.600246 | 1.576949 | 1.66 |
| C1 20 | 1.229247 | 1.121892 | 1.116103 | 1.306388 | 0.806885 | 1.121329 |
| C2 0 | 2.268761 | 2.532674 | 2.534146 | 2.654382 | 1.994572 | 1.999939 |
| C2 20 | 2.354474 | 2.593779 | 2.346234 | 2.346854 | 2.609862 | 2.611173 |
| C3 0 | 2.299302 | 2.632095 | 2.301761 | 2.548818 | 2.210633 | 2.550873 |
| C3 20 | 2.348576 | 2.764261 | 2.349029 | 2.589457 | 2.741537 | 2.590208 |
| C4 0 | 2.240059 | 2.419192 | 2.248569 | 2.689393 | 2.203098 | 2.690795 |
| C4 20 | 2.260429 | 2.688419 | 2.256579 | 2.717788 | 2.698784 | 2.701194 |
| C5 0 | 2.115539 | 2.531707 | 2.126832 | 2.513708 | 1.755615 | 2.126832 |
| C5 20 | 1.536378 | 2.625237 | 1.532278 | 2.554989 | 2.582742 | 2.552843 |

**Figure 5B**

| 6hr | Epi | R1 | R2 | R3 | R4 | R5 | C1 | C2 | C3 | C4 | C5 |
| --- | --- | --- | --- | --- | --- | --- | --- | --- | --- | --- | --- |
|  | 1.090771 | 1.041026 | 1.785571 | 2.183547 | 2.097975 | 2.210721 | 1.305097 | 1.986053 | 2.089075 | 1.825998 | 2.015034 |
|  | 1.058061 | 1.389246 | 2.177147 | 2.230422 | 2.309903 | 2.575101 | 1.293982 | 1.56193 | 1.763497 | 1.6299 | 1.638229 |
|  | 1.323755 | 1.457606 | 1.733417 | 2.323491 | 2.150481 | 1.604511 | 1.426114 | 2.138627 | 1.959933 | 1.908385 | 1.827621 |
|  | 1.264531 | 1.289133 | 1.637737 | 2.015047 | 2.132706 | 2.12817 | 1.495355 | 1.453072 | 1.959933 | 1.908385 | 1.827621 |
|  |  |  |  |  |  |  |  |  |  |  |  |
| 24hr | Epi | R1 | R2 | R3 | R4 | R5 | C1 | C2 | C3 | C4 | C5 |
|  | 1.323216 | 1.511969 | 1.520295 | 1.967048 | 2.210253 | 2.380447 | 1.431656 | 1.475153 | 1.959329 | 1.978733 | 1.981036 |
|  | 1.341026 | 1.517728 | 1.057107 | 1.789981 | 2.211551 | 2.176716 | 1.439903 | 2.171597 | 2.139225 | 2.117524 | 1.8161 |
|  | 1.08186 | 1.075623 | 1.490815 | 1.642093 | 2.200465 | 2.115686 | 1.169308 | 2.334702 | 2.25251 | 2.224231 | 2.287382 |
|  | 1.361546 | 1.1893 | 1.560331 | 1.36961 | 1.539177 | 1.397056 | 1.471794 | 2.334702 | 2.25251 | 2.224231 | 2.287382 |

**Figure 6B**

| 6hr | Epi | R1 | R2 | R3 | R4 | R5 | C1 | C2 | C3 | C4 | C5 |
| --- | --- | --- | --- | --- | --- | --- | --- | --- | --- | --- | --- |
|  | 0.669817 | 1.361401 | 1.965742 | 1.984412 | 1.874556 | 1.930303 | 1.060471 | 1.793964 | 1.864856 | 1.869325 | 1.950029 |
|  | 0.996942 | 0.995667 | 1.703450 | 2.025917 | 1.914807 | 2.046300 | 1.202239 | 1.763500 | 1.900580 | 1.906378 | 1.952640 |
|  | 0.791118 | 1.428906 | 2.049483 | 2.007080 | 1.932653 | 1.946009 | 1.335411 | 1.884632 | 1.947468 | 1.804300 | 1.465630 |
|  | 0.833483 | 0.934436 | 1.850697 | 2.157303 | 2.320000 | 2.267611 | 0.940825 | 2.165766 | 2.105743 | 2.210030 | 2.002430 |
|  |  |  |  |  |  |  |  |  |  |  |  |
| 24hr | Epi | R1 | R2 | R3 | R4 | R5 | C1 | C2 | C3 | C4 | C5 |
|  | 0.805459 | 1.168371 | 2.046793 | 2.035861 | 2.063555 | 2.018820 | 1.241186 | 1.715120 | 1.929237 | 2.123990 | 2.199102 |
|  | 1.087320 | 1.244139 | 2.008524 | 2.297041 | 2.124350 | 2.389996 | 1.294801 | 1.274042 | 1.194119 | 1.603875 | 1.630532 |
|  | 1.102449 | 1.421893 | 1.610859 | 2.008740 | 2.019077 | 2.061235 | 1.219993 | 1.389658 | 1.585952 | 1.501469 | 1.870626 |
|  | 1.501097 | 1.375295 | 2.121441 | 2.051649 | 2.062653 | 2.104905 | 1.596714 | 1.415124 | 1.681237 | 1.699969 | 1.945414 |

**Figure 7B**

| R | 44.9521508 | 34.2076697 | 24.824242 | 34.0136294 | 21.7017883 |
| --- | --- | --- | --- | --- | --- |
| E | 19.9576448 | 38.3469804 | 21.6428838 | 33.9561136 | 33.6961385 |
| C | 18.5563056 | 19.1106815 | 17.4584679 | 23.0322545 | 7.95753359 |

**Figure 7E**

| rostral 6hr | rostral 24hr | epicenter 6hr | epicenter 24hr | caudal 6hr | caudal 24hr |
| --- | --- | --- | --- | --- | --- |
| 71.47058824 | 156.0064935 | 136.2858486 | 111.3457207 | 96.764706 | 123.69186 |
| 100.3235394 | 160.0649351 | 92.64707365 | 107.1227477 | 118.37653 | 125.14535 |
| 139.4153047 | 121.5116279 | 88.69413184 | 152.6954976 | 96.964752 | 64.21875 |
| 86.76470588 | 142.5872093 | 60.88232615 | 140.8471564 | 85.670561 | 86.09375 |
| 90.29411765 | 137.0056497 | 89.02349024 | 139.4954819 | 81.011739 | 83.376025 |
|  | 142.2740113 |  | 128.2003012 |  | 74.410861 |
