# Supplementary material for: Rostro-caudal different energy metabolism leading to differences in degeneration in spinal cord injury
Source: Brain Commun. 2021 Mar 28;3(2):fcab058. doi: 10.1093/braincomms/fcab058 (PMC8066884; doi:10.1093/braincomms/fcab058)
Supplement: fcab058_Supplementary_Data [file fcab058_supplementary_data.zip › Supplementary_figures.pdf]

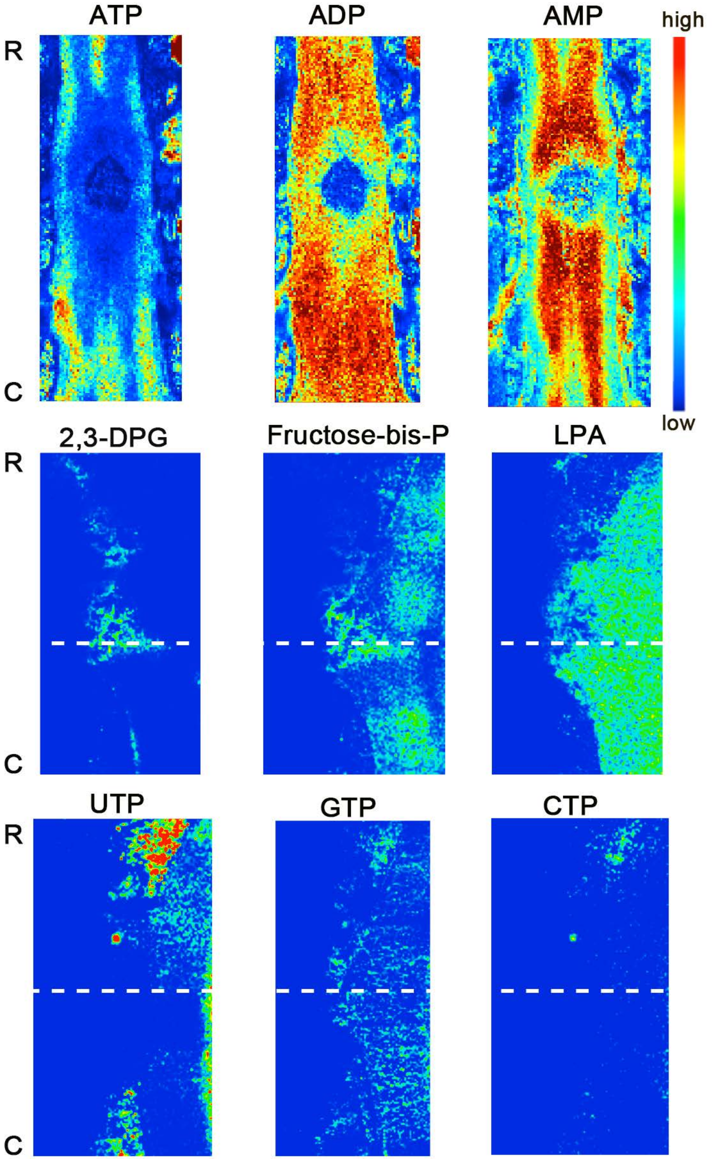

ATP
ADP
AMP
high
low
R
C
2,3-DPG
Fructose-bis-P
LPA
R
C
UTP
GTP
CTP
R
C

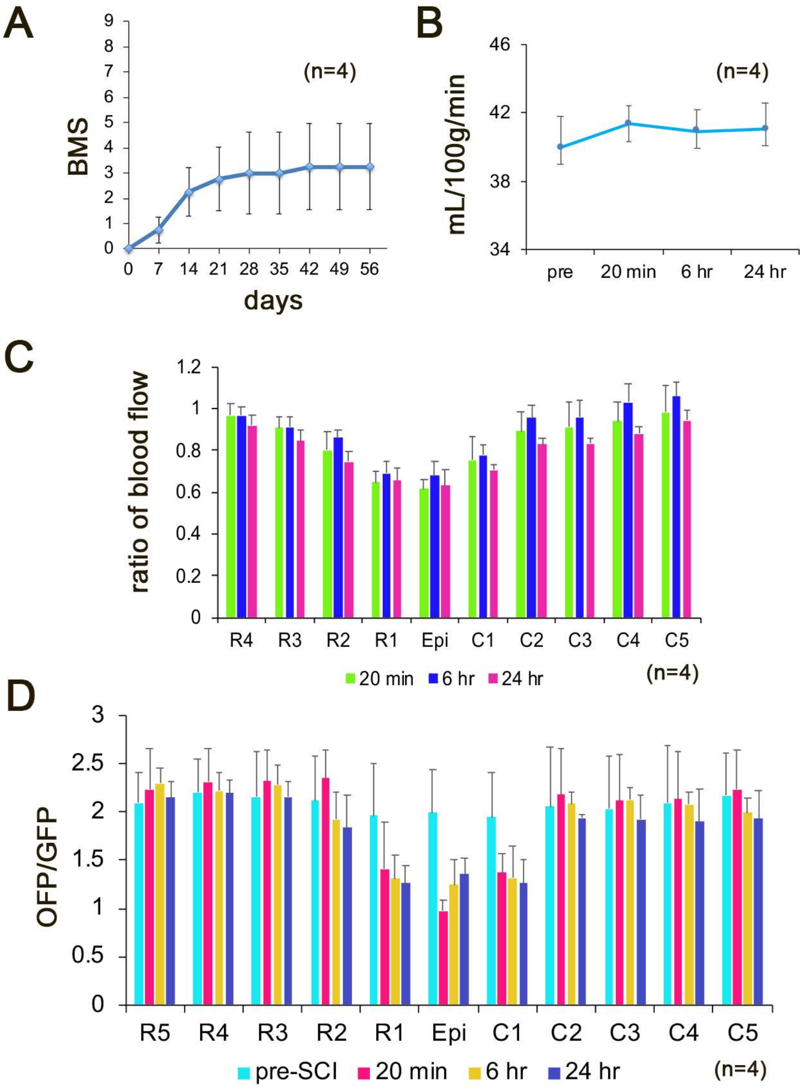
A
BMS
days
(n=4)
0
1
2
3
4
5
6
7
8
9
0
7
14
21
28
35
42
49
56
B
mL/100g/min
(n=4)
34
38
42
46
pre
20 min
6 hr
24 hr
C
ratio of blood flow
0
0.2
0.4
0.6
0.8
1
1.2
R4
R3
R2
R1
Epi
C1
C2
C3
C4
C5
20 min
6 hr
24 hr
(n=4)
D
OFP/GFP
0
0.5
1
1.5
2
2.5
3
R5
R4
R3
R2
R1
Epi
C1
C2
C3
C4
C5
pre-SCI
20 min
6 hr
24 hr
(n=4)

Supplement Figure 1. The upper panels show the coronal views. The dashed white line indicates the level of the epicenter. UTP, uridine triphosphate; GTP, guanosine triphosphate; CTP, cytidine triphosphate; 2,3-DPG, 2,3-Diphosphoglyceric acid; Hexose-P, hexose phosphate; *Fructose 1,6-bisphosphate*, fructose-bis-P; LPA, lysophosphatidic acid. R, rostral. C, caudal.

Supplement Figure 2. (A) Basso Mouse Scale score until eight weeks after SCI. (B) Blood flow values at 5-mm rostral from the epicenter before SCI (pre-SCI) and 20 min, 6 h, and 24 h after SCI. (C) Blood flow at R/C1, 2, 3, 4, and 5. (D) Intracellular ATP level before SCI (pre-SCI) and 20 min, 6 h, and 24 h after SCI. Data were analyzed using the Kruskal-Wallis test along with Dunn's post hoc test. Error bars represent the mean ± SD (n=4).
